# Biochemical Modulation of Lipid Pathway in Microalgae *Dunaliella* sp. for Biodiesel Production

**DOI:** 10.1155/2015/597198

**Published:** 2015-06-03

**Authors:** Ahmad Farhad Talebi, Masoud Tohidfar, Seyedeh Mahsa Mousavi Derazmahalleh, Alawi Sulaiman, Azhari Samsu Baharuddin, Meisam Tabatabaei

**Affiliations:** ^1^Microbial Biotechnology Department, Semnan University, Semnan 35131-19111, Iran; ^2^Microbial Biotechnology and Biosafety Department, Agricultural Biotechnology Research Institute of Iran (ABRII), Karaj 31359-33151, Iran; ^3^Biofuel Research Team (BRTeam), Karaj 31438-44693, Iran; ^4^Genomics Department, Agricultural Biotechnology Research Institute of Iran (ABRII), Karaj 31359-33151, Iran; ^5^Tropical Agro-Biomass (TAB) Group, Faculty of Plantation and Agrotechnology, Universiti Teknologi MARA (UiTM), 40450 Shah Alam, Malaysia; ^6^Faculty of Engineering, Universiti Putra Malaysia (UPM), 43400 Serdang, Malaysia

## Abstract

Exploitation of renewable sources of energy such as algal biodiesel could turn energy supplies problem around. Studies on a locally isolated strain of *Dunaliella* sp. showed that the mean lipid content in cultures enriched by 200 mg L^−1^ myoinositol was raised by around 33% (1.5 times higher than the control). Similarly, higher lipid productivity values were achieved in cultures treated by 100 and 200 mg L^−1^ myoinositol. Fluorometry analyses (microplate fluorescence and flow cytometry) revealed increased oil accumulation in the Nile red-stained algal samples. Moreover, it was predicted that biodiesel produced from myoinositol-treated cells possessed improved oxidative stability, cetane number, and cloud point values. From the genomic point of view, real-time analyses revealed that myoinositol negatively influenced transcript abundance of *AccD* gene (one of the key genes involved in lipid production pathway) due to feedback inhibition and that its positive effect must have been exerted through other genes. The findings of the current research are not to interprete that myoinositol supplementation could answer all the challenges faced in microalgal biodiesel production but instead to show that “there is a there there” for biochemical modulation strategies, which we achieved, increased algal oil quantity and enhanced resultant biodiesel quality.

## 1. Introduction

Commercial and industrial microalgae cultivation is of growing interest for numerous applications, including production of food, fertilizer, bioplastics, and pharmaceuticals, as well as algal fuel [1, 2]. Among algal species, the microalga* Dunaliella *spp. are known as the best commercial natural source for the production of* cis*-*β*-carotene. Besides, several biochemical and genetical engineering investigations have demonstrated that different species of* Dunaliella *spp. are capable of accumulating significant amounts of valuable compound such as proteins, glycerol, and also lipids [3]. As a result, they are not only a promising feedstock for biofuel production, for example, biodiesel [4], but also could potentially be used for different biotechnological processes such as foreign proteins expression and *β*-carotene production at industrial level [5]. In order to fully and economically exploit these organisms, however, some existing barriers for their large-scale cultivation should be overcome. On such basis, low cost and high quality biomass production is of great significance. To achieve such goal for biodiesel production, for instance, the following strategies could be taken into consideration in order to boost biomass productivity (BP) and volumetric lipid productivity (LP) [6].

(A) Introduction of new genes into algal cells by implementation of genetic engineering techniques stimulates some key metabolic pathways and consequently improves the energy production phenotypes in green microalgae [7] and ultimately produces biofuel at lower cost. However, despite the fact that the underlying principles laid out in this strategy are reasonable, there has been little achievement made to date. For instance, the very first genetic engineering attempts in order to increase microalgal lipid content (LC) by upregulating the first major steps of fatty acid synthesis through overexpression of acetyl-CoA carboxylase (*ACCase*) and malic enzyme were to some extent effective (12% increase in LC) [8]. Blocking-off competing pathways may also enhance the lipid accumulation in the cells. In an attempt, Picataggio and coworkers [9] blocked *β*-oxidation in* C. tropicalis* by knocking out the genes encoding acyl-CoA oxidase. Despite their expectation, it was observed that the growth of the cells was adversely affected. Therefore, enhanced lipid accumulation was rarely reported through overexpression of relevant enzymes and/or intermediate products such as FAs because of emerging a secondary rate-limiting step. Overall, given the existing obstacles facing gene transformation projects such as biosafety rules, correct introduction, and maintenance of transgenes, this strategy should not be considered as the first resort.

(B) The second strategy is the optimization of nutrient formulations [10, 11]. There have been reports that development of optimum growth condition variables [12, 13] has increased the lipid pool in some microalgal strains [14, 15]. Provision of biogenic elements, mainly nitrogen, is one of the main factors affecting algal metabolism. The change in the carbon/nitrogen ratio in a media is known to result in a change in the metabolism direction. In many algae species, increases in this ratio could contribute significantly to the accumulation of neutral lipids, mainly triacylglycerol [16–18]. On the other hand, unfortunately the conditions required for optimal production of algal biomass are different from those of lipid production; consequently, decreasing the cost through growth condition optimization approaches is not only time- and money-consuming but also, in most cases, will not significantly improve lipid accumulation [16].

(C) The last strategy would be biochemical engineering approaches, in which a variety of different plant growth regulators (PGR) could be used to support cell growth. It is generally assumed that the genetic background of a respective algal species would determine the composition of the produced lipids but the lipid amount is mainly a response to the growth conditions [19]. To channel metabolic flux generated in photobiosynthesis into lipid biosynthesis, implementation of some PGRs, vitamins, and lipid precursors could lead to an increase in total catabolism activation and lipid accumulation.

Myoinositol, as one of the nine stereoisomers of inositol, is classified as a member of the vitamin B complex and is required for the cell growth as well as other significant biological processes. Myoinositol was first used by Jacquiot [20] in order to favor bud formation and retard necrosis in elm when supplied at 20–1000 mg L^−1^; however, the proliferation of the callus was not improved. Letham [21] reported that myoinositol acts as second messengers in the primary action of auxins in plants and its interaction with cytokinin stimulates cell division in carrot phloem explants [22]. Moreover, inositol is also known as a precursor for phospholipids such phosphatidylinositol (PI) in the cells. In an investigation, it was found out that inositol addition into the growth media of yeast led to changes in transcript abundance of over 100 genes, namely, the UAS_ino_-containing genes. Many of these genes encode enzymes involved in lipid metabolism [23, 24]. For instance, in yeast, the expression of* ACCase* gene, a key gene in the synthesis of long chain fatty acids, is stimulated by inositol and choline [25].

The present study was set to investigate myoinositol-driven modulation of LP and quality (fatty acids (Fas) profile) in cultures of local* Dunaliella *sp. strain isolated from the north coast of Persian Gulf. Nile red staining using microplate fluorescence reading as well as epifluorescent microscopic and flow cytometer analyses was used to monitor the effect of the myoinositol supplementation on the algal cells. Moreover, real-time PCR analysis was performed to look into the impact of myoinositol on one of the key genes involved in lipid synthesis.

## 2. Methods

### 2.1. Strains Cultivation

A marine strain (*Dunaliella *sp.) isolated from Bandar Lengeh, a port city on the northern coast of the Persian Gulf, was used in this study. Green colonies were transferred into new flasks containing a media named Lake Media and was kept at 20°C and a constant (24 : 0) 3klux photon flux of white and red LED lamps [27]. The ingredients of Lake Media, developed during the course of the present study, are included: Lake salt sediment (60 g L^−1^), NPK fertilizer (2 g L^−1^), and FeSO_4_ (0.05 g L^−1^). The media's pH was set at 7.5 and the samples were constantly shaking at 120 rpm.

Beside the strain locally isolated in this study, one other local strain,* D. salina* (generously provided by Dr. Shariati, Isfahan University), and two standard stains, CCAP 19/18 and UTEX 200, purchased from the Culture Collection of Algae and Protozoa (Sams Research, Scotland), and University of Texas at Austin, (Austin, USA), respectively, were also included in the experiments. All the strains were cultivated in the Lake Media under the above-mentioned conditions. During the cultivation period, growth kinetic parameters were recorded for all the strains in triplicate. Data comparison was then carried out using the ANOVA test. The calculated growth parameters included BP, LC, and LP (mg L^−1^ day^−1^). For BP determination, algal suspensions were centrifuged (3000 g, 10 min) and the wet weights were determined gravimetrically. LP was calculated according to the following equation:

(1) $$\begin{array}{c} LP=BP\times LC. \end{array}$$

Besides, to investigate the effect of myoinositol inclusion on lipid metabolism and biodiesel properties, only the Persian Gulf strain was used and cultured in the Lake Media supplemented with 0, 50, 100, and 200 mg L^−1^ myoinositol.

### 2.2. Molecular Identification

Isolation of total DNA content from the studied strains was carried out by using the DNeasy plant minikit (QIAGENE, Germany). Species-specific oligonucleotides, namely, MA1 and MA3 (without any restriction site), corresponding to the conserved regions of 5′ and 3′ termini were used to amplify 18S rDNA gene. PCR reactions were performed according to the method described by Olmos and coworkers [26]. The molecular weights of PCR-amplified products were calculated and confirmed using a gel documentation system. PCR amplicons were purified using the PCR purification kit (Roche) according to the manufacturer's instructions. Then, the purified products were sequenced by Macrogen Company (Korea). Using BLAST software, the obtained sequences were compared with those deposited in NCBI GenBank as 18S rDNA and ITS regions of different* Dunaliella* species.

A neighbor-joining tree was constructed using the software MEGA version 4. Evolutionary distances were computed using the maximum composite likelihood model. For analysis, 1000 bootstrap replicates were performed to assess the statistical support for the tree. Phylogenetic studies included* Chlamydomonas pumilio*, as the outgroup.

### 2.3. RNA Extraction, Reverse Transcription, and Real-Time PCR Analysis

To extract RNA from algal cells, 50 mg wet biomass (28-day old culture) was harvested by centrifugation at 2000 ×g for 10 min. Separated algae cells in microtube were disrupted by glass rod in liquid nitrogen, then 500 *μ*L TRIzol reagent (Invitrogen, Carlsbad, CA, USA) was added, and RNA was extracted according to the manufacturer's instructions. Concentrations and purity of the total RNA extracted were measured spectrophotometrically. The extracted RNAs were treated with DNase to eliminate genomic DNA contamination and continuously reverse transcription (RT) was carried out using QuantiTect Reverse Transcription kit (Qiagen). Real-time PCR was performed using a Bio-Rad iCycler iQ real-time PCR.

The first rate-limiting step in the FA biosynthesis pathway was regulated by ACCase [29]. Since AccD subunit overexpression leads to boosted ACCase activity, the effect of myoinositol on* AccD* transcript accumulation was studied. Gene-specific primer pairs of* AccD* and a housekeeping gene used for PCR are listed in Table 5. The 18S rRNA transcript was used to normalize the results by eliminating variations in the quantity and quality of mRNA and cDNA. A reaction mixture for each PCR run was prepared with the SYBR Green supermix (Bio-Rad). The cycle parameters consisted of one cycle of 100 s at 95°C and then 35 cycles of 30 s at 95°C followed by 30 s at 62°C and 20 s at 72°C. Data were collected at the end of each extension step. The relative quantification of gene expressions among the treatment groups was analyzed by the 2^−ΔΔCt^ method [30], where Ct is the cycle number at which the fluorescent signal rises statistically above the background.

### 2.4. Fluorescent Measurement of Microalgal Neutral Lipids

The intracellular neutral lipid distribution in microalgal cells was examined by staining the cells harvested from 500 *μ*L cell suspension, with 300 *μ*L working solution (1 *μ*M) of Nile red fluorescent dye (Sigma-Aldrich, St. Louis, MO) diluted in Hanks and 20 mM Hepes buffer (HHBS), pH 7. The stock solution was first prepared by dissolving Nile red in anhydrous DMSO (1 mM). The cells were incubated at 37°C for 10 min and protected from light. To remove the Nile red working solution from the cells, the cells were washed and resuspended in HHBS. Cells were examined by an epifluorescent microscope Leica DMRXA with a Nikon (DXM 1200) digital camera (Nikon, Tokyo, Japan) with an excitation wavelength of 486 nm; the emission was measured at 570 nm, following the method of Cooney et al. [28].

For fluorescence-based quantification of the accumulated lipids, a high-throughput technique reported by Chen and coworkers [31] was followed with some modifications. The base procedure was performed in two models: (1) measuring total emitted fluorescence by staining same volume of cell suspension for different treatments (cells at the lag stationary phase), which would quantify total produced lipid volumetrically; (2) measuring emitted fluorescence by staining same cell number (10^5^ cells) for different treatment, which would quantify stored lipid in cells. Both procedures involved staining the cell suspensions with Nile red as mentioned above. Fluorescence was measured on a Varian 96-well plate spectrofluorometer. The excitation and emission wavelengths of 522 and 628 nm were selected, based on a previously published report [28].

### 2.5. Flow Cytometry Study

To figure out the duration after which myoinositol supplementation would lead to an improving effect on lipid synthesis, flow cytometry analysis was used. To investigate that, 7- and 35-day cell suspensions, at linear growth phase and lag stationary phase, respectively, were analyzed. Cells were stained with working Nile red solution as explained earlier. The optical system used in the EPICS XL flow cytometer collects yellow light (575 band-pass filters) in the FL2 channel, corresponding to the Nile red fluorescence in a neutral lipid matrix. To remove nonalgal particles, chlorophyll fluorescence characteristics were considered. Approximately 10,000 cells were analyzed using a log amplification of the fluorescence signal. Unstained cells were used as autofluorescence control. The data used was the arithmetic mean of all cytometric events (10,000 cells) in 3 repeats and two independent experiments [32].

### 2.6. Oil Extraction and Fatty Acid Profile by Gas Chromatography (GC) Analysis

All cultures, by three replications, were allowed to grow for 35 days to reach the lag stationary phase and then cells were harvested for lipid profile analysis. By this means, the effects of growth phase on the total LC and FA profile were minimized [28]. LC reported as percentage of the total biomass (%dwt) was determined based on the Bligh and Dyer method [33] and was obtained in triplicate for the different strains. Data comparison was then carried out using the ANOVA test.

For GC analysis, the direct transesterification method was used based on the procedure reported by Lepage and Roy with minor modifications [34]. The samples containing hexane and FAME were used for GC analysis. The FAs determination was carried out on a Varian CP-3800 GC (Varian Inc., Palo Alto, CA) equipped with a CP-Sil88 fused silica column (100 m, 0.25 mm I.D., film thickness 0.25 *μ*m). The oven temperature was programmed as our previous report [6]. Fatty acid peeks were identified by comparison of the retention time with FAME standards.

### 2.7. Biodiesel Properties by Bioprospection

Biodiesel quality parameters, that is, oxidation stability, cold performance (cloud point), and combustion characteristics (cetane number), were estimated based on the fact that they are directly influenced by the molecular structure of FAME like the carbon chain length and the amount and/or position of double bonds [35]. Oxidation stability parameters estimated were iodine value (IV), APE, and BAPE. All these parameters were calculated based on the FAME profile using empirical equations as detailed in previous report [36].

## 3. Result and Discussion

### 3.1. Identification

**Figure 1:**
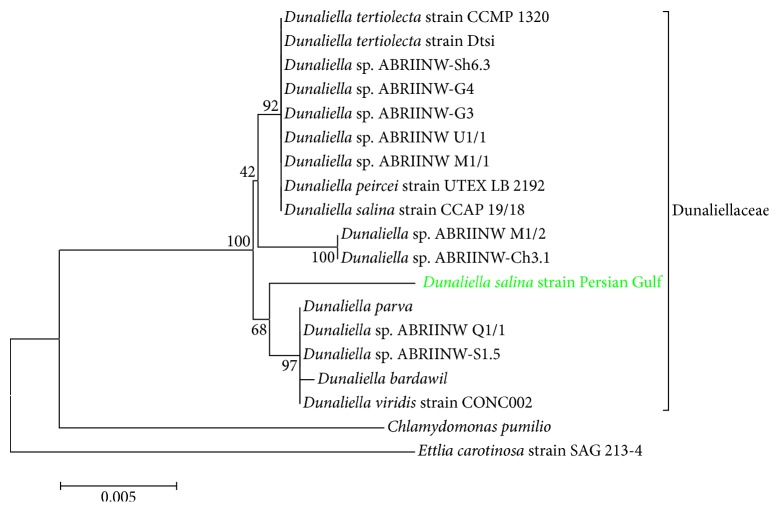
NJ bootstrap consensus tree showing the relationships among* Dunaliella salina* (Persian Gulf) and other standard and Iranian strains. Bootstrap values were calculated over 1000 replicates.* Chlamydomonas pumilio* and* Ettlia carotinosa* were considered as outgroups.

Molecular identification and discrimination of living eukaryotic organisms based on the comparison of 18S rDNA gene sequences are a promising method and have been frequently used for molecular identification of different species of* Dunaliella* [26, 37, 38]. Hereby, the chromosomal DNA of 18S using the forward primer MA1 and reverse primer MA3 was amplified. A single band representing the amplified DNA product of 1.7 kb was recorded, which could be used in the intron sizing method for the identification of* Dunaliella* genus [39]. The sequence was aligned with 18 different strains whose 18S sequences were previously submitted at NCBI. The 18S sequence of the* Dunaliella *sp., Persian Gulf strain, was registered in NCBI database with an accession number of KF477384. This sequence exhibited high similarities to other members of Dunaliellaceae. The highest similarity was observed with* Dunaliella* sp. ABRIINWQ1/1 at 98%, which was isolated from an ancient saline lake located in the middle of Iran plateau (Qum Salt Lake). This finding, coupled with the morphological features of the isolated strain (Figure 1), confirmed that the saline water isolate strain is a member of* Dunaliella* genus. The cells lack a cell wall and a well-developed apical papilla and two equal-long (25.0–30.0 *μ*m) and smooth flagella also uphold this identification.

As for the size, number, and position of introns of the 18S rDNA gene, in the* Dunaliella* sp., three types of 18S rDNA structure have been reported by Olmos et al. [40]: no-intron genes with a size of ~1770 bp, one-intron gene with a size of ~2170 bp, and finally the genes with two introns and with a size of ~2570 bp. Interestingly, using intron-sizing method in this genus was applied to an indicator for selection of hyperproducing species [40]. Based on this method, the Persian Gulf strain belonged to the first group with no intron in the 18S rDNA gene. This strain never turned red, indicating that *β*-carotene was not hyperproduced by this strain. In order to properly explore the similarity observed among the obtained 18S sequences and, moreover, to determine their phylogenetic relationship, a phylogenetic tree was reconstructed using the neighbor-joining (NJ) method. NJ result along with the bootstrap coefficients (replication ×1000) is depicted in Figure 1. In this classification, Persian Gulf strain was under the family of Dunaliellaceae, close to Qum strain (Q1/1) and hypo-*β*-carotene producing strains. As observed in the figure, the Persian Gulf strain is situated close to* D. parva *and* D. viridis *which also confirmed the finding of the intron-sizing method, since these two strains have low *β*-carotene production capacity as well but can grow in hypersaline environments [40].

### 3.2. Studying the Algal Species Using Growth Parameters

Since the intracellular LC and FAs profile of microalgae are affected by both culture conditions and growth phase, all of the studied strains were cultivated under the same conditions (flasks containing Lake Media were kept at 20°C and a constant (24 : 0) 3klux photon flux of white and red LED lamps). Sole influence of myoinositol addition on lipid metabolism was also investigated under the same conditions as well. All cultures were harvested after reaching the stationary phase. The results of LC, BP, and LP have been presented in Table 1.

BP value slightly fluctuated for all the strains between 0.12 and 0.15 g L^−1^ day^−1^, except in case of* D. salina* (Shariati) where significantly lower value of 0.05 g L^−1^ day^−1^ was recorded. Myoinositol addition into the* Dunaliella *sp. (Persian Gulf) culture caused a constant but nonmeaningful decrease in the BP value proportional to the increasing concentrations of myoinositol. The cells grown in the highest concentration of myoinositol (200 mgL^−1^) had 20% less BP in comparison with those grown in myoinositol free culture (0.12 and 0.15 g L^−1^ day^−1^, resp.).

The lowest amount of LC was obtained for* D. salina* (Shariati) followed by* Dunaliella *sp. (Persian Gulf), (18.9 and 22% dwt, resp.). In contrast, the highest LC values were also achieved for* Dunaliella* sp. (Persian Gulf) when myoinositol was added to the culture media. More specifically, the mean value of LC for cultures enriched by 200 mg L^−1^ myoinositol was around 33%. This record represents 50% increase in oil accumulation in comparison to the control. Similarly, the LP values were classified into four significantly different groups. The highest LP value belonged to the myoinositol treatment group. These findings revealed the efficiency of biochemical engineering strategies. This was also reported in another biochemical modulation study on the algae* C. sorokiniana* using 0.1% tryptophan supplementation. The authors recorded 57.28% enhancement in LP just 4 days after the treatment [41]. Their findings as well as those of the present study could in a way confirm the promising role of biochemical modulation when combined with selection of proper strains in enriching lipids quantity and consequently in biodiesel production.

Overall, LP, as an indicator of the produced oil in terms of both volume and time, showed a sharp increase after myoinositol addition. This parameter has been reported as a suitable variable to evaluate algal species potential for biodiesel production [42]. This would highlight the positive impact of myoinositol on increasing algal potential for producing biodiesel.

### 3.3. Integrated Growth and Lipid Production Using a Novel Algal Media

Generally, a two-stage cultivation is considered for algal lipid production: growth stage in which high N concentration is used to achieve the highest possible BP followed by the second stage where N-starvation is imposed to encourage lipid production [8]. James and coworkers [43] observed that when nitrogen was deprived for 4 days, LC increased for all the studied strains of* C. reinhardtii*. In their study, using such 2-stage cultivation strategy, the total FAs of the wild-type strains increased 1.3- and 1.4-fold. However, this would increase the cost and consequently deteriorates the economic aspect of the algal fuel production scenario in comparison with single-stage cultivation. On the other hand, it has been well documented that algal cultivation in a media with decreased nitrogen content results in a decline in biomass content [44]. This is ascribed to the fact that N-starvation leads to decreased duration of the exponential growth phase [45]. Therefore, providing high N content throughout the cultivation period while encouraging lipid production through other alternatives could play a significant role in achieving an economic algal biodiesel. In light of that in the present study, a new media named Lake Media, capable of encouraging high BP, was formulized using 2 g L^−1^ of NPK fertilizer, as a nitrogen source, and a considerable quantity of natural lake salt (60 g L^−1^). By considering the different cost of by-product by* Dunaliella* sp. grown in various batch culture media, it is obviously clear that implementation of newly developed media in this research, Lake Media, provides a golden opportunity in large-scale cultivation of this microalgae and final production economically. In another study on this media, implementation of Lake Media declined the costs of carotenoid production to just 0.0029 USD mg^−1^ which is 200% lower than the standard media (Johnson and Olmos Media) and it compensates the lower cell density obtained by Lake Media (unpublished data).

At the same time and with simultaneity of BP increase, LC was also successfully encouraged in this media by myoinositol inclusion as lipid precursor. As a result, the normally used biphasic algal cultivation and lipid production were simplified into a single-phased process in which the combination of the Lake Media and myoinositol met all the requirements of the cells for growth and lipid synthesis simultaneously. This strategy could lead to decreasing the final cost of produced biodiesel.

### 3.4. Impact of Myoinositol on Acetyl-coA Carboxylase: A Key Gene in Lipid Synthesis

Quantification of the relative-fold change in mRNA levels of the AccD gene 28 days after myoinositol supplementation, in the treated sample in comparison with the control group, was conducted using the real-time PCR analysis. The 2^−ΔΔCt^ value decreased from 0.25 for the 100 mg L^−1^ myoinositol to 0.2 for 200 mg L^−1^. As presented in Table 2,* AccD* gene exhibited decreasing responses to myoinositol supplementation. In fact, myoinositol resulted in a 75–80% decrease in* AccD* transcript abundance as compared to the control sample. This decrease could be explained as follows: supplementation of myoinositol as a lipid precursor caused an increase in lipid production and accumulation and this in turn resulted in a feedback inhibition, downregulation of the genes involved in lipid synthesis including* AccD*. This explanation is in line with the findings of Al-Feel who investigated the impact of inositol supplementation on lipid production while monitoring the response of another key gene involved in lipid production pathway, acetyl Co-A carboxylase (ACC_1_) [25]. They reported that* ACC*_*1*_ was repressed due to inositol supplementation but returned to near basal expression level by steady state.

Therefore, it could be concluded that inositol and its stereoisomer, myoinositol, exert their positive effect on lipid production through other genes involved in lipid production pathway and not the* AccD* and* ACC*_*1*_ genes. In case of the* AccD* gene, this could also be confirmed by the fact that inositol stimulates transcription of a series genes which have a conserved domain in their promoter called UAS_ino_ (inositol-sensitive upstream activating sequence) element. This sequence is absent in the* AccD's* promoter. As for the* ACC*_*1*_, despite the presence of this element, moderate variation in the expression of this gene by inositol supplementation was reported [24, 46].

On the other hand, based on the model presented by Thomas and Fell concerning regulation of enzymatic pathways, many enzymes are involved in controlling the rate of a reaction and alteration of one alone may have a small impact [47]. Therefore, one could point out that myoinositol could have influenced many metabolic processes, rather than targeting a single enzyme in a pathway, and as a result increased the total lipid accumulation. However, increased lipid production and accumulation and consequent feedback inhibition resulted in the downregulation of the key genes involved in lipid production pathway, that is,* AccD* and* ACC*_*1*_.

Such hypothesis could be supported by the findings of a number of studies revealing that the extent of involved genes upregulation was not reflected in the fatty acid (FA) profile/content [48].

### 3.5. Proposed Molecular Mechanisms for Lipid Increase by Myoinositol Supplementation

In the present study, the total lipid accumulation was sharply increased by 50% in response to myoinositol implementation which could be attributed to myoinositol role in simultaneously increasing lipid storage and membrane lipids [49]. Previously, a survey on* Saccharomyces cerevisiae* showed a swift 5-6-fold increase in cellular membrane phosphatidylinositol (PI) content in response to inositol inclusion. This rise in PI content seems to be positively correlated with FA synthesis [50]. More specifically, inositol leads to higher production of negatively charged PI and consequently lower negative surface charge of the membrane. On the other hand, the distribution of long-chain acyl-CoA molecules in membrane and activity of* ACCase* are both controlled by negative surface charge, and therefore myoinositol supplementation would increase the capacity of the membrane for incorporation of long-chain acyl CoAs and consequently enhance the cellular FFA synthesis [51].

**Figure 2:**
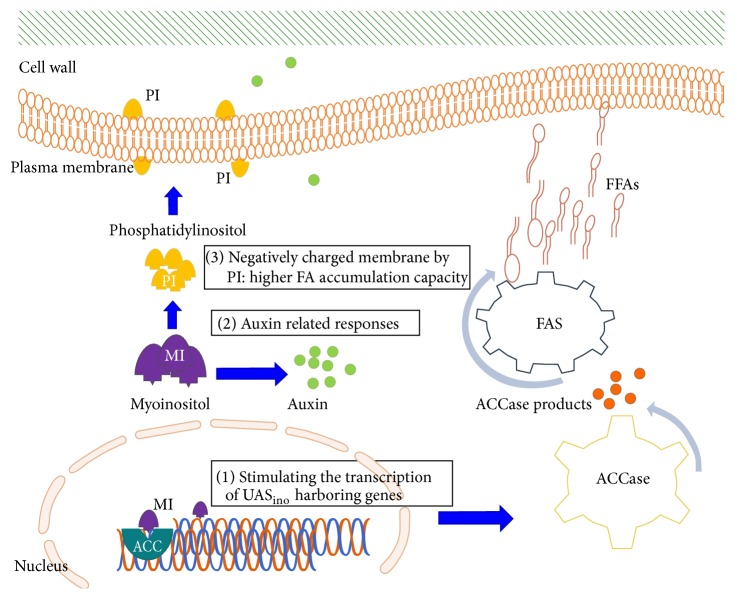
Proposed mechanisms for the lipid-promoting effects of myoinositol (MI) in algal cells, (1) through stimulating the transcription of the responsive genes (e.g., ACC) harboring inositol-sensitive upstream activating sequence (UAS_ino_) element in their promoters which could in turn positively impact FA synthesis (FAS), (2) through auxin-related responses, and (3) by increasing membrane negative charge regulated by phosphatidylinositol (PI).

Although mechanisms describing the growth-promoting effect of myoinositol on algal cell have not been described precisely, their growth-promoting effects through plant growth regulators (phytohormones) have been previously pointed out [52, 53]. In fact, stimulation of signaling pathways by phytohormones plays a vital role in plant responses to environmental changes. Among phytohormones, auxin which is involved in plant growth and development [54] is regulated by the concentration of phosphoinositides, which are mainly synthesized from myoinositol [50]. In a study, Arroussi and coworkers managed to increase initial lipid content of* D. tertiolecta* (24%) to 38% and 43% after addition of auxin at 0.5 and 1 mg/L, respectively [55]. Therefore, one possible explanation for the lipid-promoting effect of myoinositol in* D. salina* could be attributed to the action of auxins. Moreover, myoinositol plays a key role in cell membrane charge alteration (by PI) which could consequently increase the membrane capacity for FFAs accumulation. Finally, myoinositol also stimulates the transcription of the responsive genes (e.g., ACC) harboring UAS_ino_ element in their promoter which could in turn positively impact FA synthesis (FAS). The proposed mechanisms for the lipid-promoting effects of myoinositol are presented in Figure 2.

### 3.6. Fluorescence Microscopic Study of Neutral Lipids

**Figure 3:**
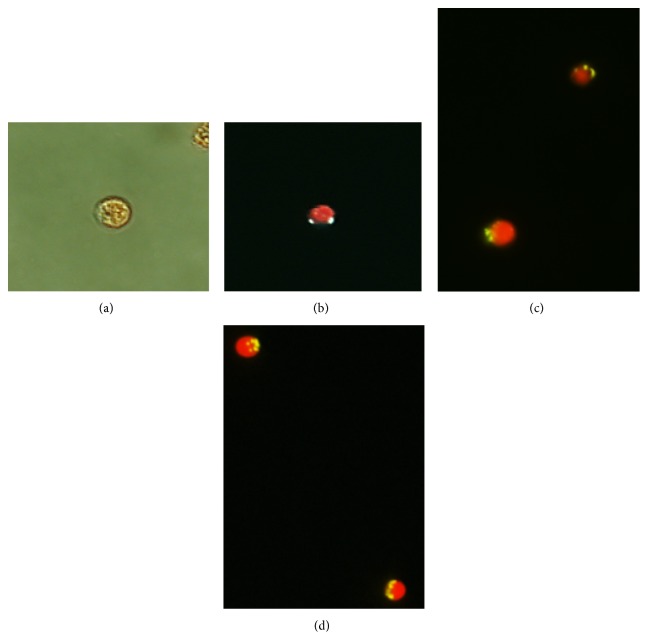
Epifluorescent microphotographs (magnification ×40) of microalgae stained with fluorochrome Nile red. Neutral lipids in cells are seen as lighter colored drops. (a) Bright field image and fluorescence image; (b) control; (c) 100 mg L^−1^ myoinositol supplementation; (d) 200 mg L^−1^ myoinositol supplementation. Microphotographs were taken using a Leica DMRXA compound light microscope with a Nikon (DXM 1200) digital camera, a band-pass filter with an excitation range of 450–490 nm, and a long-pass suppression filter with an edge wavelength of 515 nm.

Nile red has been widely used to screen wild and mutant variation to explore new candidates for biodiesel production from microalgae to dinoflagellate [56, 57]. In this study, the liposoluble fluorescence probe Nile red was used to visualize neutral lipids in the cells. As shown in Figure 3, the lipid bodies appear as yellow fluorescing circular organelles, while the red background fluorescence is attributed to chlorophyll autofluorescence. The highest LC, as determined by Nile red staining, was observed in cells treated by 200 mg L^−1^ myoinositol. In these cells, small drops of neutral lipids were seen dispersed throughout the cytoplasm (Figure 3), while cells with no myoinositol addition in the media were comparatively poor in terms of neutral LC during the lag stationary phase and showed limited number of small lipid bodies in the cells.

### 3.7. Quantitative Fluorescence Measurement of Neutral Lipids

#### 3.7.1. Microplate Fluorometry

**Figure 4:**
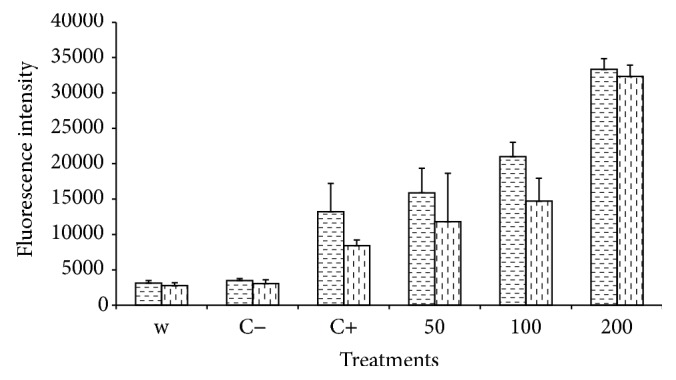
Fluorescence emission of Nile red-stained microalgae. The excitation and emission wavelengths for fluorescence measurement were at 522 and 628 nm, respectively. The cell density of the suspensions used for analysis was 10^5^ cell mL^−1^. Nile red staining was conducted based on the procedures described by Cooney et al. [28]. Data were the mean values of three replicates (vertical dashed lines: same volume; horizontal dashed lines: same cell number). C− and C+ represent nonstained and stained cell with no myoinositol inclusion, respectively.

Result of total lipid measurement using microplate fluorometryin the same volume of the cell suspension was summarized in Figure 4. The fluorescence intensity dramatically increased in the samples treated by the increasing amount of myoinositol. More specifically, implementation of 100 mg L^−1^ myoinositol sharply increased the recorded fluorescence by 59% and further by 152% for 200 mg L^−1^ myoinositol in comparison with that of the control. All treatments were significantly different (at *P* < 0.01).

Effect of cell concentration on the fluorescence of neutral lipids was also taken into consideration by staining the same cell concentration (10^5^ cells mL^−1^) for all the investigated samples to measure the relative amount of lipid stored in same number of cells. Similar to the results of total lipid measurement in cell suspensions, myoinositol was proven to stimulate single cells to accumulate more lipid in their cytoplasm. 200 mg L^−1^ myoinositol implementation led to 284% raise in the emitted fluorescence. A significant difference between the three studied levels of myoinositol (50, 100, and 200 mg L^−1^) could be seen in the same cell number mode (120% increase in fluorescence emission for 100 and 200 mg L^−1^). This was also confirmed by total extracted lipid data based on which 50, 100, and 200 mg L^−1^ myoinositol supplementation caused 13, 23, and 50% increase in LC, respectively.

#### 3.7.2. Flow Cytometry

Flow cytometry, in conjunction with microplate fluorometry, represents an invaluable tool for screening and exploiting high lipid-producing microalgae strains. In this study, lipid accumulation was studied using flow cytometry 7 and 35 days after myoinositol supplementation. The results obtained revealed that on day 35 the mean fluorescence for the control was 199.6, whereas this value was at 225.65, 263.6, and 283.1 (13, 32, and 42% increase in comparison with the control), for the cells treated by 50, 100, and 200 mg L^−1^ myoinositol, respectively.

**Figure 5:**
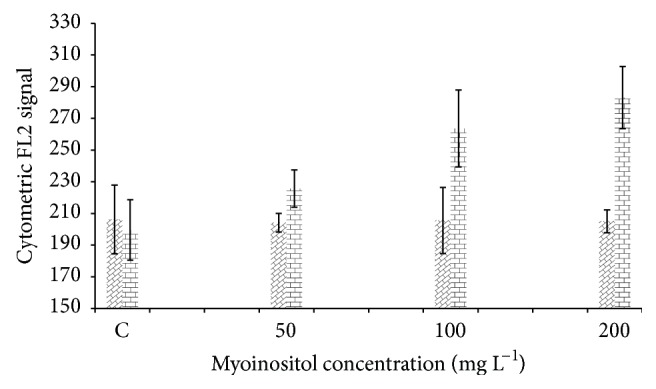
Variation of cytometric signal (FL2: yellow fluorescence, *λ* = 575 nm) in cells stained with Nile red (*Dunaliella *sp.). Horizontal and diagonal bricks represent the sample treated by myoinositol for 35 and 7 days, respectively.

Moreover, the effect of exposure time to myoinositol and its relation with cell growth phase and lipid accumulation were also studied by comparing the mean fluorescence on days 7 and 35. The results clearly confirmed that the positive effect of myoinositol supplementation on lipid production is visible in the stationary phase when algal cells have already completed their growth or, in other words, the emitted fluorescence values recorded in the young algal cells (on the day 7) were not significantly different among the treatments (Figure 5).

Overall, there was a clear correlation between the fluorometry (microplate fluorescence and flow cytometry) results and those of LC gravimetrical determination (Figures 4 and 5 and Table 1). Therefore, fluorometry techniques could be suggested as powerful and high-throughput alternative analytical tools to monitor the effect of chemicals and biological molecules on lipid production and accumulation in algal cells.

### 3.8. The Role of Myoinositol Treatment on Algal FAs Profiles

Fatty acid methyl ester (FAME) profiles for different algal strains studied in this study are summarized in Table 3. It has been frequently reported that 16–18 carbon chain FAs are dominant in algal cells [6, 28, 58]. The same observation was made in this study as well. In all the strains investigated, the omega-3 fatty acid, linolenic acid (18 : 3) made up the highest portion of the FAME profile. Persian Gulf strain grown in the Lake Media with no myoinositol enrichment was found to have the highest percentage for this FA (>44%) while the lowest record for 18 : 3 of around 26% was also observed in the same strain, but in presence of myoinositol. On the contrary, myoinositol inclusion (200 mg L^−1^) led to increased percentages of monounsaturated FAs (MUFA) that is, palmitoleic acid (16 : 1) and oleic acid (18 : 1) by 4.6- and 0.4-folds, respectively. Moreover, in case of linoleic acid (18 : 2), low concentration of myoinositol (100 mg L^−1^) considerably increased percentage of this FA, while 200 mg L^−1^ myoinositol implementation led to a significant decrease in 18 : 2 accumulation. Overall, MUFA were increased continuously by myoinositol addition in the media while PUFA were felt down vice versa.

These findings were similar to those of studies in which N-starvation was applied to enhance lipid accumulation in microalgae. Gurr and Harwood [59] reported relative accumulations in 18 : 1 and 18 : 2, accompanied by a decrease in 18 : 0 acid when N-starvation was used. They explained that N-starvation promoted the desaturation pathways, beginning with delta-9 desaturase. In a similar study using N-starvation on* Botryococcus braunii*, an increase in the content of SFAs (up to 76.8%) and a substantial decrease in the PUFA content (up to 6.8%) were observed [19]. It could be concluded that myoinositol might also encourage the desaturation pathways.

### 3.9. Estimation of Biodiesel Properties

The impact of myoinositol on key biodiesel quality parameters such as allylic position equivalents (APE) and bisallylic position equivalents (BAPE), cetane number (CN), and cloud point (CP) was also investigated. The BAPE and APE values are effective means of predicting oxidative instability of biodiesel. The highest BAPE and APE values were measured for the control Persian Gulf (no myoinositol addition), while the lowest values were recorded for UTEX200 strain (Table 4). This could be explained by the highest and lowest levels of unsaturated FAs in particular 18 : 3 in the control Persian Gulf and UTEX200 strain, respectively. A decreasing trend for BAPE and APE values and consequently higher oxidative stability were observed when algal cells (Persian Gulf strain) were treated by myoinositol. For instance, BAPE decreased by 26% at myoinositol concentration of 200 mg L^−1^ (Table 4).

CN indicates the combustion quality of diesel fuels including biodiesel. In other words, the higher this value is, the easier it would be to start a standard diesel engine. An increase in this parameter was observed where algal cells were treated by myoinositol. Slight improvements in the estimated CP values were also recorded. Overall, it was shown that inclusion of myoinositol led to improved fuel properties.

In a different study Ngangkham et al. [41] also strived to improve* C. sorokiniana* oil quality for biodiesel production but different treatments from that of this study were applied. In particular, they reported reduced level of PUFA and consequent increased oxidative stability of the produced biodiesel. In conclusion and based on the findings of Ngangkham et al. [41] and those of the present investigation, biochemical engineering treatments could be regarded as efficient strategies for directed improvement of algal oil quality and resultant biodiesel based on a particular climate condition.

## 4. Conclusion

The present study was set to evaluate the effects of myoinositol on a locally isolated Persian microalgae strain,* Dunaliella *sp., with a specific focus on LP and biodiesel quality based on FA composition. Inclusion of myoinositol (200 mg L^−1^) in the media improved the total lipid accumulation (up to 50%) and biodiesel quality parameters, that is, APE, BAPE, CN, and CP. Hypothetically, myoinositol treatment led to increased auxin and PI accumulation in the cells and consequently more negatively charged membranes. This in turn resulted in increased FFA synthesis and lipid accumulation. Biochemical modulation strategies should still be progressively considered in hope of finding more efficient and economically feasible strategies leading to more viable production systems.

## Figures and Tables

**Table 1:** Biomass productivity, lipid content, and lipid productivity of the microalgae strains.

Strains	Parameters		
	Biomass productivity (BP, g L^−1^ day^−1^)	Lipid content (LC, %dwt)^*^	Volumetric lipid productivity Pb × LC × 1000 (LP, mg L^−1^ day^−1^)
*Dunaliella salina* (Shariati)	0.05^A^	18.9 ± 1.1^A^	10.26 ± 0.4^A^
*D*. *salina* (UTEX)	0.15^C^	24 ± 1.3^B^	36.48 ± 0.6^C^
*D*. *salina* (CCAP19/18)	0.14^B^	25.1 ± 0.7^B^	35.14 ± 0.2^C^
*Dunaliella* sp. (Persian Gulf)	0.15^C^	22 ± 2^B^	33 ± 0.3^B^
1^#^	0.14^B^	25 ± 0.5^C^	36.15 ± 0.9^C^
2^#^	0.14^B^	27 ± 0.5^C^	38.6 ± 0.4^D^
3^#^	0.12^B^	33 ± 1^D^	39.3 ± 0.6^D^

^*^All cultures harvested after reaching the stationary phase and LC was determined based on the Bligh and Dyer method [33]. Data are expressed as mean ± SD (*n* = 3). Means of BP, LC, and LP are compared using one-way ANOVA and ones with different letter are significantly different (at *P* < 0.05).

^#^1, 2, and 3 representing 50, 100, and 200 mg L^−1^ myoinositol implementation in Persian Gulf strain, respectively.

**Table 2:** Real-time PCR analysis of gene expression. Values were normalized against 18S rRNA as housekeeping gene and represent the relative mRNA expression (mean standard error) of three replicate cultures.

Treatment	C_T AccD_	C_T 18S_	ΔC_T.treat_	ΔC_T.control_	ΔΔC_T_	2^−ΔΔ Ct^
Control	26.7 ± 0.46	21.2 ± 1.1	−5.5	—	—	—
50 mg	27.19 ± 0.33	19.38 ± 0.5		−7.81	−2.31	0.2
100 mg	26.35 ± 0.41	18.87 ± 0.85	—	−7.48	−1.98	0.25
200 mg	30.27 ± 0.62	22.43 ± 0.72	—	−7.83	−2.33	0.2

**Table 3:** Types of fatty acids produced and properties of algal oil.

Strain	Fatty acid (%)							SFA	MUFA	PUFA	SFA/USFA
	16 : 0	16 : 1	18 : 0	18 : 1	18 : 2	18 : 3	20 : 1				
*Dunaliella* sp. (Persian Gulf)	9.19 ± 1.2	0.80 ± 0.8	4.27 ± 0.9	22.51 ± 0.7	3.84 ± 0.4	44.31 ± 2.1	1.42 ± 0.2	13.47	24.74	48.15	0.16
*D*. *salina* (Shariati)	12.02 ± 2.1	4.45 ± 0.2	1.91 ± 1.2	23.67 ± 1.6	2.28 ± 1.1	40.36 ± 2.2	1.40 ± 0.2	13.93	29.52	42.65	0.16
*D*. *salina* (UTEX200)	16.34 ± 1.4	1.04 ± 0.9	6.43 ± 1.2	19.58 ± 1.1	6.76 ± 1.2	27.71 ± 2.5	2.28 ± 0.3	22.77	22.89	34.47	0.28
*D*. *salina* (CCAP19/18)	15.87 ± 1.8	ND^+^	6.14 ± 1.3	21.39 ± 2.1	15.92 ± 1.6	23.95 ± 1.9	ND	22.01	21.39	39.87	0.26
1^*^	7.05 ± 0.9	6.25 ± 0.3	1.55 ± 0.7	22.95 ± 0.8	12.15 ± 0.3	37.66 ± 0.4	1.12 ± 0.6	8.60	30.32	49.81	0.10
2^*^	7.75 ± 0.6	5.04 ± 0.8	1.42 ± 0.5	25.27 ± 1.9	18.58 ± 1.4	26.91 ± 1.9	ND	9.17	30.31	45.48	0.11
3^*^	8.41 ± 0.8	4.52 ± 1.1	2.14 ± 0.3	32.03 ± 2.2	13.58 ± 1.3	27.24 ± 1.4	ND^−^	10.55	36.55	40.82	0.12

^*^1, 2, and 3 representing 50, 100, and 200 mg L^−1^ myoinositol implementation in Persian Gulf strain, respectively.

^+^Not detected.

^−^Non identified Fas which are around 10%.

**Table 4:** Comparison of the estimated properties of algal biodiesel from cells treated with myoinositol.

Strains	Biodiesel properties			
	CN	CP	BAPE	APE
*Dunaliella* sp. (Persian Gulf)	43.75	−0.16	92.47	118.82
*D*. *salina* (Shariati)	45.65	1.33	83.01	108.96
*D*. *salina* (UTEX200)	55.36	3.60	62.17	88.51
*D*. *salina* (CCAP19/18)	52.96	3.36	63.83	101.14
1^*^	42.27	−1.28	87.47	122.57
2^*^	47.61	−0.91	72.39	116.23
3^*^	47.16	−0.57	68.06	113.66

^*^1, 2, and 3 representing 100 and 200 mg L^−1^ myoinositol implementation in Persian Gulf strain, respectively.

**Table 5:** Sequences of primer pairs used in real-time PCR.

Primer name	Sequence
18S rRNA (forward)	5′-CAGACACGGGGAGGATTGACAGATTGAGAG-3′
18S rRNA (reverse)	5′-GCGCGTGCGGCCCAGAACATC-3′
*AccD* (forward)	5′-AAGACGCACAAGAACGAACAG-3′
*AccD* (reverse)	5′-AACTACAGAGCCCATACTTCCC-3′

## Abbreviations

ACCase: Acetyl-CoA carboxylase
APE: Allylic position equivalents
BAPE: Bisallylic position equivalents
CN: Cetane number
CP: Cloud point
*D*.: *Dunaliella*
FA: Fatty acid
FAME: Fatty acid methyl ester
IV: Iodine value
LC: Lipid content
BP: Biomass productivity
LP: Lipid productivity
MSFA: Monosaturated FAs
MUFA: Monounsaturated FAs
PGR: Plant growth regulator
PI: Phosphatidylinositol
SFA: Total saturated fatty acids.

## References

[1] Menetrez M. Y. (2014). Meeting the US renewable fuel standard: a comparison of biofuel pathways. *Biofuel Research Journal*.

[2] Bux F. (2014). The potential of using wastewater for microalgal propagation. *Biofuel Research Journal*.

[3] Specht E., Miyake-Stoner S., Mayfield S. (2010). Micro-algae come of age as a platform for recombinant protein production. *Biotechnology Letters*.

[4] Beetul K., Bibi Sadally S., Taleb-Hossenkhan N., Bhagooli R., Puchooa D. (2014). An investigation of biodiesel production from microalgae found in Mauritian waters. *Biofuel Research Journal*.

[5] Georgianna D. R., Hannon M. J., Marcuschi M. (2013). Production of recombinant enzymes in the marine alga *Dunaliella tertiolecta*. *Algal Research*.

[6] Talebi A. F., Mohtashami S. K., Tabatabaei M. (2013). Fatty acids profiling: a selective criterion for screening microalgae strains for biodiesel production. *Algal Research*.

[7] Tabatabaei M., Tohidfar M., Jouzani G. S., Safarnejad M., Pazouki M. (2011). Biodiesel production from genetically engineered microalgae: future of bioenergy in Iran. *Renewable & Sustainable Energy Reviews*.

[8] Talebi A. F., Tohidfar M., Bagheri A., Lyon S. R., Salehi-Ashtiani K., Tabatabaei M. (2014). Manipulation of carbon flux into fatty acid biosynthesis pathway in *Dunaliella salina* using AccD and ME genes to enhance lipid content and to improve produced biodiesel quality. *Biofuel Research Journal*.

[9] Picataggio S., Rohrer T., Deanda K. (1992). Metabolic engineering of *Candida tropicalis* for the production of long-chain dicarboxylic acids. *Bio/Technology*.

[10] Talebi A. F., Tabatabaei M., Mohtashami S. K., Tohidfar M., Moradi F. (2013). Comparative salt stress study on intracellular ion concentration in marine and salt-adapted freshwater strains of microalgae. *Notulae Scientia Biologicae*.

[11] Olivieri G., Marzocchella A., Andreozzi R., Pinto G., Pollio A. (2011). Biodiesel production from *Stichococcus* strains at laboratory scale. *Journal of Chemical Technology and Biotechnology*.

[12] Elumalai S., Prakasam V. (2011). Optimization of abiotic conditions suitable for the production of biodiesel from *Chlorella vulgaris*. *Indian Journal of Science and Technology*.

[13] Sasi D., Mitra P., Vigueras A., Hill G. A. (2011). Growth kinetics and lipid production using *Chlorella vulgaris* in a circulating loop photobioreactor. *Journal of Chemical Technology and Biotechnology*.

[14] Work V. H., Radakovits R., Jinkerson R. E. (2010). Increased lipid accumulation in the *Chlamydomonas reinhardtii* sta7-10 starchless isoamylase mutant and increased carbohydrate synthesis in complemented strains. *Eukaryotic Cell*.

[15] Mallick N., Mandal S., Singh A. K., Bishai M., Dash A. (2012). Green microalga *Chlorella vulgaris* as a potential feedstock for biodiesel. *Journal of Chemical Technology and Biotechnology*.

[16] Piorreck M., Baasch K.-H., Pohl P. (1984). Biomass production, total protein, chlorophylls, lipids and fatty acids of freshwater green and blue-green algae under different nitrogen regimes. *Phytochemistry*.

[17] Chu W.-L., Phang S.-M., Goh S.-H. (1996). Environmental effects on growth and biochemical composition of *Nitzschia inconspicua* Grunow. *Journal of Applied Phycology*.

[18] Thompson G. A. (1996). Lipids and membrane function in green algae. *Biochimica et Biophysica Acta: Lipids and Lipid Metabolism*.

[19] Zhila N. O., Kalacheva G. S., Volova T. G. (2005). Effect of nitrogen limitation on the growth and lipid composition of the green alga *Botryococcus braunii* Kütz IPPAS H-252. *Russian Journal of Plant Physiology*.

[20] Jacquiot C. (1951). Action of meso-inositol and of adenine on bud formation in the cambium tissue of *Ulmus campestris* cultivated in vitro. *Comptes Rendus de l'Académie des Sciences*.

[21] Letham D. S. (1966). Regulators of cell division in plant tissues—II. A cytokinin in plant extracts: isolation and interaction with other growth regulators. *Phytochemistry*.

[22] Watanabe K., Tanaka K., Asada K., Kasai Z. (1971). The growth promoting effect of phytic acid on callus tissues of rice seed. *Plant and Cell Physiology*.

[23] Santiago T. C., Mamoun C. B. (2003). Genome expression analysis in yeast reveals novel transcriptional regulation by inositol and choline and new regulatory functions for Opi1p, ino2p, and ino4p. *The Journal of Biological Chemistry*.

[24] Jesch S. A., Zhao X., Wells M. T., Henry S. A. (2005). Genome-wide analysis reveals inositol, not choline, as the major effector of Ino2p-Ino4p and unfolded protein response target gene expression in yeast. *Journal of Biological Chemistry*.

[25] Al-Feel W., DeMar J. C., Wakil S. J. (2003). A *Saccharomyces cerevisiae* mutant strain defective in acetyl-CoA carboxylase arrests at the G2/M phase of the cell cycle. *Proceedings of the National Academy of Sciences of the United States of America*.

[26] Olmos J., Paniagua J., Contreras R. (2000). Molecular identification of *Dunaliella* sp. utilizing the 18S rDNA gene. *Letters in Applied Microbiology*.

[27] Droop M. R. (1954). A note on the isolation of small marine algae and flagellates for pure cultures. *Journal of the Marine Biological Association of the United Kingdom*.

[28] Cooney M. J., Elsey D., Jameson D., Raleigh B. (2007). Fluorescent measurement of microalgal neutral lipids. *Journal of Microbiological Methods*.

[29] Madoka Y., Tomizawa K.-I., Mizoi J., Nishida I., Nagano Y., Sasaki Y. (2002). Chloroplast transformation with modified *accD* operon increases acetyl-CoA carboxylase and causes extension of leaf longevity and increase in seed yield in tobacco. *Plant and Cell Physiology*.

[30] Livak K. J., Schmittgen T. D. (2001). Analysis of relative gene expression data using real-time quantitative PCR and the 2^−∆∆*C*_*T*_^ method. *Methods*.

[31] Chen W., Zhang C. H., Song L., Sommerfeld M., Qiang H. (2009). A high throughput Nile red method for quantitative measurement of neutral lipids in microalgae. *Journal of Microbiological Methods*.

[32] Lee Y.-H., Chen S.-Y., Wiesner R. J., Huang Y.-F. (2004). Simple flow cytometric method used to assess lipid accumulation in fat cells. *Journal of Lipid Research*.

[33] Bligh E. G., Dyer W. J. (1959). A rapid method of total lipid extraction and purification. *Canadian Journal of Biochemistry and Physiology*.

[34] Lepage G., Roy C. C. (1986). Direct transesterification of all classes of lipids in a one-step reaction. *The Journal of Lipid Research*.

[35] Sarin A., Arora R., Singh N. P., Sarin R., Malhotra R. K., Kundu K. (2009). Effect of blends of Palm-Jatropha-Pongamia biodiesels on cloud point and pour point. *Energy*.

[36] Talebi A. F., Tabatabaei M., Chisti Y. (2014). BiodieselAnalyzer: a user-friendly software for predicting the properties of prospective biodiesel. *Biofuel Research Journal*.

[37] Olmos-Soto J., Paniagua-Michel J., Contreras R., Trujillo L. (2002). Molecular identification of *β*-carotene hyper-producing strains of dunaliella from saline environments using species-specific oligonucleotides. *Biotechnology Letters*.

[38] Raja R., Hema S., Balasubramanyam D., Rengasamy R. (2007). PCR-identification of *Dunaliella salina* (Volvocales, Chlorophyta) and its growth characteristics. *Microbiological Research*.

[39] Hejazi M. A., Barzegari A., Gharajeh N. H., Hejazi M. S. (2010). Introduction of a novel 18S rDNA gene arrangement along with distinct ITS region in the saline water microalga Dunaliella. *Saline Systems*.

[40] Olmos J., Ochoa L., Paniagua-Michel J., Contreras R. (2009). DNA fingerprinting differentiation between-carotene hyperproducer strains of Dunaliella from around the world. *Saline Systems*.

[41] Ngangkham M., Ratha S. K., Prasanna R. (2012). Biochemical modulation of growth, lipid quality and productivity in mixotrophic cultures of *Chlorella sorokiniana*. *SpringerPlus*.

[42] Rodolfi L., Zittelli G. C., Bassi N. (2009). Microalgae for oil: strain selection, induction of lipid synthesis and outdoor mass cultivation in a low-cost photobioreactor. *Biotechnology and Bioengineering*.

[43] James G. O., Hocart C. H., Hillier W. (2011). Fatty acid profiling of *Chlamydomonas reinhardtii* under nitrogen deprivation. *Bioresource Technology*.

[44] An J.-Y., Sim S.-J., Lee J. S., Kim B. W. (2003). Hydrocarbon production from secondarily treated piggery wastewater by the green alga *Botryococcus braunii*. *Journal of Applied Phycology*.

[45] Brenckmann F., Largeau C., Casadevall E., Berkaloff C. (1985). Influence de la nutrition azotéesur la croissance et la production des hydrocarbures de l’algue unicellulaire *Botryococcus braunii*. *Energy from Biomass*.

[46] Schuller H. J., Hahn A., Troster F., Schutz A., Schweizer E. (1992). Coordinate genetic control of yeast fatty acid synthase genes *FAS1* and *FAS2* by an upstream activation site common to genes involved in membrane lipid biosynthesis. *EMBO Journal*.

[47] Thomas S., Fell D. A. (1998). The role of multiple enzyme activation in metabolic flux control. *Advances in Enzyme Regulation*.

[48] Lei A., Chen H., Shen G., Hu Z. H., Chen L., Wang J. (2012). Expression of fatty acid synthesis genes and fatty acid accumulation in *Haematococcus pluvialis* under different stressors. *Biotechnology for Biofuels*.

[49] Gaspar M. L., Hofbauer H. F., Kohlwein S. D., Henry S. A. (2011). Coordination of storage lipid synthesis and membrane biogenesis. *Journal of Biological Chemistry*.

[50] Gaspar M. L., Aregullin M. A., Jesch S. A., Henry S. A. (2006). Inositol induces a profound alteration in the pattern and rate of synthesis and turnover of membrane lipids in *Saccharomyces cerevisiae*. *The Journal of Biological Chemistry*.

[51] Sumper M. (1974). Control of fatty acid biosynthesis by long chain acyl CoAs and by lipid membranes. *European Journal of Biochemistry*.

[52] Stevenson J. M., Perera I. Y., Heilmann I., Persson S., Boss W. F. (2000). Inositol signaling and plant growth. *Trends in Plant Science*.

[53] Luo Y., Qin G., Zhang J. (2011). D-myo-inositol-3-phosphate affects phosphatidylinositol-mediated endomembrane function in Arabidopsis and is essential for auxin-regulated embryogenesis. *Plant Cell*.

[54] Zhang S., van Duijn B. (2014). Cellular auxin transport in algae. *Plants*.

[55] El Arroussi H., Benhima R., Bennis I., El Mernissi N., Wahby I. (2015). Improvement of the potential of *Dunaliella tertiolecta* as a source of biodiesel by auxin treatment coupled to salt stress. *Renewable Energy*.

[56] Fuentes-Grünewald C., Garcés E., Rossi S., Camp J. (2009). Use of the dinoflagellate *Karlodinium veneficum* as a sustainable source of biodiesel production. *Journal of Industrial Microbiology & Biotechnology*.

[57] Doan T. T. Y., Sivaloganathan B., Obbard J. P. (2011). Screening of marine microalgae for biodiesel feedstock. *Biomass and Bioenergy*.

[58] Lang I., Hodac L., Friedl T., Feussner I. (2011). Fatty acid profiles and their distribution patterns in microalgae: a comprehensive analysis of more than 2000 strains from the SAG culture collection. *BMC Plant Biology*.

[59] Gurr M. I., Harwood J. L. (1991). *Lipid Biochemistry: An Introduction*.

